# Wastewater metagenomics in Africa: Opportunities and challenges

**DOI:** 10.1371/journal.pgph.0004044

**Published:** 2024-12-20

**Authors:** Stephen Kanyerezi, Fatma Zahra Guerfali, Abbas Abel Anzaku, Oluwasegun Adesina Babaleye, Tracey Calvert-Joshua, Julien Alban Nguinkal, Oluwaseun Paul AMOO, Chiraz Atri, Waqasuddin Khan, Iqra Saleh, M. Imran Nisar, Arthur Shem Kasambula, Koketso Morapedi, Gerald Mboowa

**Affiliations:** 1 The African Center of Excellence in Bioinformatics and Data-Intensive Sciences, the Infectious Diseases Institute, College of Health Sciences, Makerere University, Kampala, Uganda; 2 National Health Laboratories and Diagnostics Services, Central Public Health Laboratories, Ministry of Health, Kampala, Uganda; 3 Department of Immunology and Molecular Biology, School of Biomedical Sciences, College of Health Sciences, Makerere University, Kampala, Uganda; 4 Laboratory of Transmission, Control and Immunobiology of Infections, Institut Pasteur de Tunis, University Tunis-El Manar, Tunis, Tunisia; 5 Department of Clinical Laboratory Services, Institute of Human Virology, Abuja, Nigeria; 6 Global Health and Infectious Diseases Control Institute, Nasarawa State University Keffi, Keffi, Nigeria; 7 Microbiology Department, The Center for Human Virology and Genomics, Nigerian Institute of Medical Research, Lagos, Nigeria; 8 Public Health Alliance for Genomic Epidemiology (PHA4GE), University of the Western Cape, Bellville, South Africa; 9 Department of Infectious Disease Epidemiology, Bernhard-Nocht Institute for Tropical Medicine, Hamburg, Germany; 10 African Center of Excellence for Genomics of Infectious Diseases, Redeemers University, Ede, Nigeria; 11 Pneuma Research Institute (PRI), Nigeria; 12 Department of Pediatrics and Child Health, CITRIC Center for Bioinformatics and Computational Biology, Aga Khan University, Karachi, Pakistan; 13 Incident Management Team, Ministry of Health, Kampala, Uganda; 14 Department of Natural Sciences, Institute of Health Sciences-Gaborone, Gaborone, Botswana; 15 Genformatics Centre of Excellence, Gaborone, Botswana; 16 Africa Centres for Disease Control and Prevention, African Union Commission, Addis Ababa, Ethiopia; University of St. Andrews, UNITED KINGDOM OF GREAT BRITAIN AND NORTHERN IRELAND

## Abstract

The advent of metagenomics has dramatically expanded our understanding of microbial communities, particularly through the study of wastewater, which serves as a rich source of microbial data. In Africa, wastewater metagenomics presents unparalleled opportunities for public health monitoring, antimicrobial resistance (AMR) tracking, and the discovery of new microbial species and functions. Utilizing high-throughput sequencing (HTS) technologies, this method allows for direct analysis of nucleic acids from wastewater samples, providing a cost-effective and comprehensive approach for pathogen surveillance. The potential of wastewater metagenomics in Africa is vast. It can revolutionize public health monitoring by acting as an early warning system for infectious disease outbreaks, offering near real-time data to shape effective responses. This is especially critical in densely populated urban areas with poor sanitation, where the risk of disease spread is high. Moreover, this approach enables the detection of emerging pathogens and insights into environmental health. However, the implementation of wastewater metagenomics in Africa faces several challenges. These include variability in wastewater composition due to differing local customs, limited infrastructure for sequencing and data analysis, and a shortage of bioinformatics expertise. Socio-political and ethical issues also complicate data sharing and the equitable distribution of benefits. To overcome these challenges, there is a need to enhance capacity through collaborative training, infrastructural development, and international partnerships. Investing and sustaining local genomics and bioinformatics infrastructure and expertise is crucial. Moreover, establishing robust data governance frameworks and engaging communities are essential for leveraging metagenomics to advance scientific knowledge and deliver tangible health and economic benefits. With strategic planning and collaboration, Africa can harness the transformative potential of wastewater metagenomics to improve disease surveillance, combat AMR, and foster scientific innovation, contributing significantly to sustainable development and improved quality of life.

## 1. Introduction

Africa faces a diverse array of infectious diseases caused by a wide range of pathogens, from viral infections like Lassa fever, Ebola [1] to bacterial diseases such as cholera and tuberculosis [2] anthrax and parasitic infections like malaria and schistosomiasis. In Africa, many surveillance systems are primarily based on syndromic reporting. These traditional public health surveillance systems often fail to detect pathogens early or overlook certain diseases, especially in regions with limited healthcare access and diagnostic services. Many pandemic and epidemic-prone diseases go unreported, particularly among populations with restricted access to healthcare facilities, resulting in incomplete and skewed surveillance data [3].

Wastewater acts as an extensive and unbiased reservoir of diverse microbial communities, capturing pathogens shed by both symptomatic and asymptomatic individuals, irrespective of their socioeconomic status. It does not differentiate between those who access health facilities and those who do not, thus collecting data from the entire population-encompassing both the affluent and the disadvantaged, as well as residents from urban and rural areas [4]. This makes wastewater an essential ecosystem for detecting a wide spectrum of pathogens circulating in diverse communities.

By analyzing wastewater, researchers can identify and quantify various pathogens, creating an early warning system for potential outbreaks. This method provides a comprehensive overview of public health, surpassing the limitations of traditional surveillance methods. In areas with limited healthcare infrastructure, wastewater metagenomics fills a critical gap, offering valuable insights into the epidemiology of diseases that might otherwise remain undetected.

In the African context, where a wide range of pathogens coexist and healthcare access is often inconsistent, wastewater metagenomics stands out as a powerful tool for public health surveillance. This approach enables the early detection of disease outbreaks, the tracking of infectious agents, and the monitoring of antibiotic resistance patterns across diverse populations [5]. By providing a comprehensive view of public health trends, wastewater metagenomics can guide targeted interventions, inform resource allocation, and ultimately improve health outcomes across the continent. In this paper, we examine the challenges and opportunities associated with implementing wastewater metagenomics in Africa.

## 2. Opportunities

### 2.1 Disease surveillance

**Fig 1:**
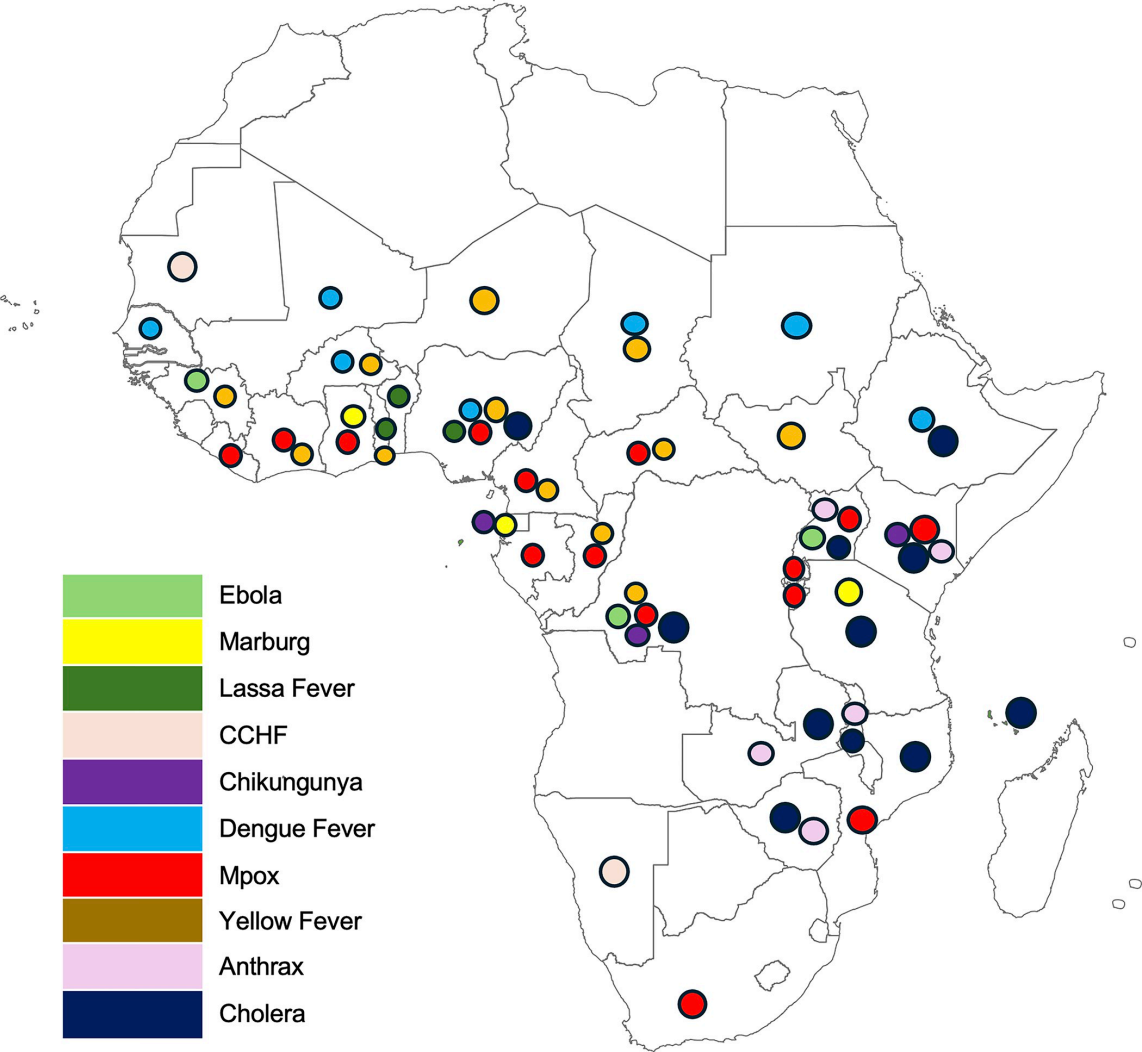
Countries in Africa that have reported at least one of the selected 10 pathogens of epidemic and pandemic potential since 2020. The base map shapefile for Africa was downloaded from: Natural Earth (http://www.naturalearthdata.com/). This work is licensed under a Creative Commons Attribution CC-BY 4.0 license.

Since 2020, Africa has faced multiple outbreaks with epidemic and pandemic potential, in addition to COVID-19, as shown in **Fig 1**. These situations underscore the necessity for early detection and rapid response to prevent widespread outbreaks and save lives.

Effective disease surveillance allows health authorities to allocate resources efficiently, tailoring public health interventions to mitigate the impact of infectious diseases. Surveillance of emerging and reemerging infectious diseases is an ongoing process that requires the systematic sampling, collection, analysis, interpretation and dissemination of information on disease outbreak for public health intervention [6]. The implementation of wastewater-based surveillance (WBS) in Africa could significantly transform infectious disease detection, particularly in regions where traditional healthcare systems are weak.

The application of metagenomics to wastewater enables the detection of pathogens from the large communities, providing more precise public health data than traditional surveillance methods. This is particularly crucial for Sub-Saharan Africa (SSA), where zoonotic diseases and emerging infections pose persistent threats. Additionally, WBS has proven effective in monitoring the spread of MPXV. A study conducted in 22 Canadian wastewater treatment plants (WWTPs) demonstrated that WBS can serve as a valuable public health tool by offering early warnings about the emergence of clinical cases [7]. Despite the global recognition and the significant role of WBS in monitoring the COVID-19 pandemic, this method remains underutilized in SSA. This underutilization could hinder the early detection, monitoring, prevention, and response to outbreaks in the region [8]. By integrating WBS into existing frameworks such as the Integrated Disease Surveillance and Response (IDSR), Africa could significantly improve its capacity to quickly detect and monitor infectious disease outbreaks. WBS could equally play a vital role in facilitating timely and targeted interventions. Over time, the implementation of wastewater metagenomics in Africa has the potential to revolutionize public health surveillance by offering a cost-effective and scalable solution to bridge the gap between inadequate healthcare systems and the need for comprehensive disease monitoring.

### 2.2. Microbial diversity studies

The study of microbial diversity in African wastewater systems using metagenomics provides critical insights into the role of these ecosystems in disease outbreaks and AMR. For instance, metagenomic analysis of Kampala’s wastewater revealed a diverse microbial community, including the identification of both prevalent pathogens like *Pseudomonas psychrophila* and less common ones such as *Klebsiella pneumoniae*. The study also detected 23 antimicrobial resistance genes (ARGs), highlighting the potential of wastewater metagenomics to uncover the diversity, abundance, and dynamics of pathogens and resistance genes in the environment [9]. This approach is invaluable for public health surveillance, enabling the early detection of emerging pathogens and ARGs that traditional methods might overlook. Metagenomics also facilitates the study of unculturable microbes and mobile genetic elements, which are crucial in the spread of AMR. Integrating metagenomics into wastewater research is vital for monitoring microbial diversity, assessing environmental impacts, and addressing global AMR challenges. These efforts are important in Africa, where unique water and sanitation conditions shape microbial diversity and influence the dynamics of antimicrobial resistance.

### 2.3. Antimicrobial resistance monitoring

WWTPs gather wastewater from diverse sources, such as hospitals and residential areas. Consequently, they process a mix of human waste that may contain a range of contaminants, including pathogens, ARGs and antibiotic-resistant bacteria (ARB) [10]. By analyzing wastewater and identifying ARGs within these contaminants, researchers and public health officials can utilize data from WWTPs to gauge the distribution and prevalence of ARBs, pathogens, and ARGs in the broader community. This information is critical for assessing public health risks and developing strategies to curb the spread of antibiotic resistance and infectious diseases [11].

WWTPs are critical for monitoring and controlling the spread of ARGs, ARB, and pathogens in communities. The rising use of antibiotics, particularly in low- and middle-income countries (LMICs), has exacerbated the issue of ARGs and ARB, underscoring the need for robust surveillance systems. While research indicates that wastewater treatment processes can reduce the presence of resistance genes and pathogenic bacteria, they do not completely eliminate them. Public health officials can utilize data from WWTPs to monitor the spread of ARGs and ARBs, aiding in the development of more informed strategies to address public health risks. Incorporating metagenomics analysis of wastewater into public health systems can significantly enhance efforts against antibiotic resistance and infectious diseases. This approach offers a cost-effective means to track the spread of resistance and tailor specific interventions. However, to fully leverage this method’s potential in safeguarding public health, continuous investment in research funding, technological advancements, and improved infrastructure is essential.

### 2.4. Environmental impact assessments: Understanding the impact of human activities on ecosystems

Wastewater metagenomics provides a potent tool for environmental impact assessments (EIA) in Africa, offering a deeper insight into how human activities influence various ecosystems. Traditional EIA methods often overlook the complexity of microbial communities and their significance in ecosystem health. However, metagenomic techniques can detect the presence and diversity of pathogens, ARGs, and other microbial indicators of pollution and ecosystem disturbances. By incorporating wastewater metagenomics into EIAs, policymakers and environmental managers can gain a more precise understanding of the anthropogenic impacts on ecosystems, leading to better-informed decision-making and more effective mitigation strategies. This method not only advances our knowledge of environmental health but also establishes a framework for monitoring and safeguarding Africa’s critical resources amid escalating urbanization and industrialization.

### 2.5. Technological advancements: Adoption and innovation in sequencing technologies and bioinformatics tools

Technological advancements present a significant opportunity for wastewater metagenomics progress in Africa. While there has been an increase in the acquisition of high throughput next-generation sequencing platforms and bioinformatics expertise following the COVID-19 pandemic in Africa, there distribution remains uneven. Advanced sequencing technologies like Illumina and nanopore sequencing are predominantly concentrated in established research institutions and bioinformatics hubs in countries like South Africa, Kenya, Nigeria, and Uganda [12–15]. Such developments have dramatically increased the speed, accuracy, and affordability of metagenomic analyses within those countries. Portable sequencing technologies, such as nanopore sequencing, offer the potential for on-site and real-time data collection, bypassing the need for advanced laboratory facilities and enabling rapid pathogen detection and response to emerging health threats [16, 17].

Advancements in cloud computing and data storage have made it easier to manage and analyze large metagenomic datasets, enabling researchers in resource-limited settings to collaborate with international experts and utilize advanced analytical tools. These technological innovations democratize access to sophisticated metagenomic techniques, empowering African scientists to conduct high-level research and contribute to global health surveillance. By harnessing these advancements, Africa can bolster its capabilities in comprehensive public health monitoring, health security, and disease control, effectively transforming wastewater metagenomics from a theoretical concept into a practical and impactful tool.

### 2.6. Utility in refugee settlements

In 2024, the Africa Center for Strategic Studies reported a record level of forced displacement in Africa due to ongoing conflicts. The report highlighted that Africa is home to the largest number of internally displaced persons (IDPs) and refugees worldwide, accounting for over 48% of the world’s IDPs and nearly one-third of the global refugee population [18]. Wastewater metagenomics offers a unique opportunity to enhance public health monitoring in refugee settlements, where traditional healthcare infrastructure is often inadequate. These settlements, characterized by high population density and limited sanitation facilities, are hotspots for infectious diseases. Conventional surveillance methods may miss significant health threats due to underreporting and limited access to diagnostic services. Wastewater metagenomics can fill this gap by providing a non-invasive, community-wide surveillance tool that captures the health status of entire populations. By analyzing waste water from refugee camps, health authorities can detect the presence of pathogens such as cholera, typhoid, and hepatitis, enabling early intervention and targeted disease control measures. This approach not only aids in the timely identification of outbreaks but also helps in monitoring the overall health trends within these vulnerable communities, ultimately contributing to better health outcomes and preventing the spread of diseases both within and beyond the settlements.

### 2.7. Novel pathogen discovery and uncovering unculturable microbes

Wastewater metagenomics presents a unique opportunity for the discovery of novel pathogens, particularly those that contribute to fevers of unknown origin in both humans and animals. Traditional diagnostic methods often fail to identify the causative agents of such fevers, especially when dealing with microbes that are difficult or impossible to culture in the laboratory settings. By analyzing the genetic material present in wastewater, researchers can uncover a wide array of microorganisms, including those that have never been cultured or characterized before. This approach not only enhances our understanding of microbial diversity but also plays a crucial role in identifying emerging pathogens that may pose significant public health risks. Moreover, the ability to detect these microbes at an early stage can inform public health interventions and help prevent outbreaks, making wastewater metagenomics a vital tool in both human and animal health surveillance in Africa.

### 2.8. One health

The One Health approach emphasizes the interconnected health of humans, animals, and the environment, advocating for coordinated efforts to tackle issues like AMR. AMR is a growing global threat, driven by factors such as the overuse of antibiotics and poor sanitation, leading to the spread of ARB throughout the ecosystem. WWTPs, particularly in densely populated or industrial areas, are key sites for both the surveillance and dissemination of ARGs. There are critical gaps in measuring AMR in hospitals, communities, animals, and the environment. SSA has the lowest antibiotic consumption (AMC) but the highest burden of AMR [19]. Furthermore, the region lacks data on comprehensive population-based surveillance of AMR [20]. Wastewater-Based Epidemiology (WBE) emerges as a powerful tool for early detection and monitoring of AMR, complementing traditional methods and offering insights into pathogen spread. Metagenomic analysis of wastewater can quantify a vast array of resistance genes, providing a cost-effective and comprehensive approach to AMR surveillance. Studies have demonstrated regional variations in ARGs prevalence, and integrating WWS into broader AMR monitoring can enhance our understanding of AMR transmission dynamics and inform strategies to mitigate its impact.

### 2.9. Hospital wastewater surveillance

**Fig 2:**
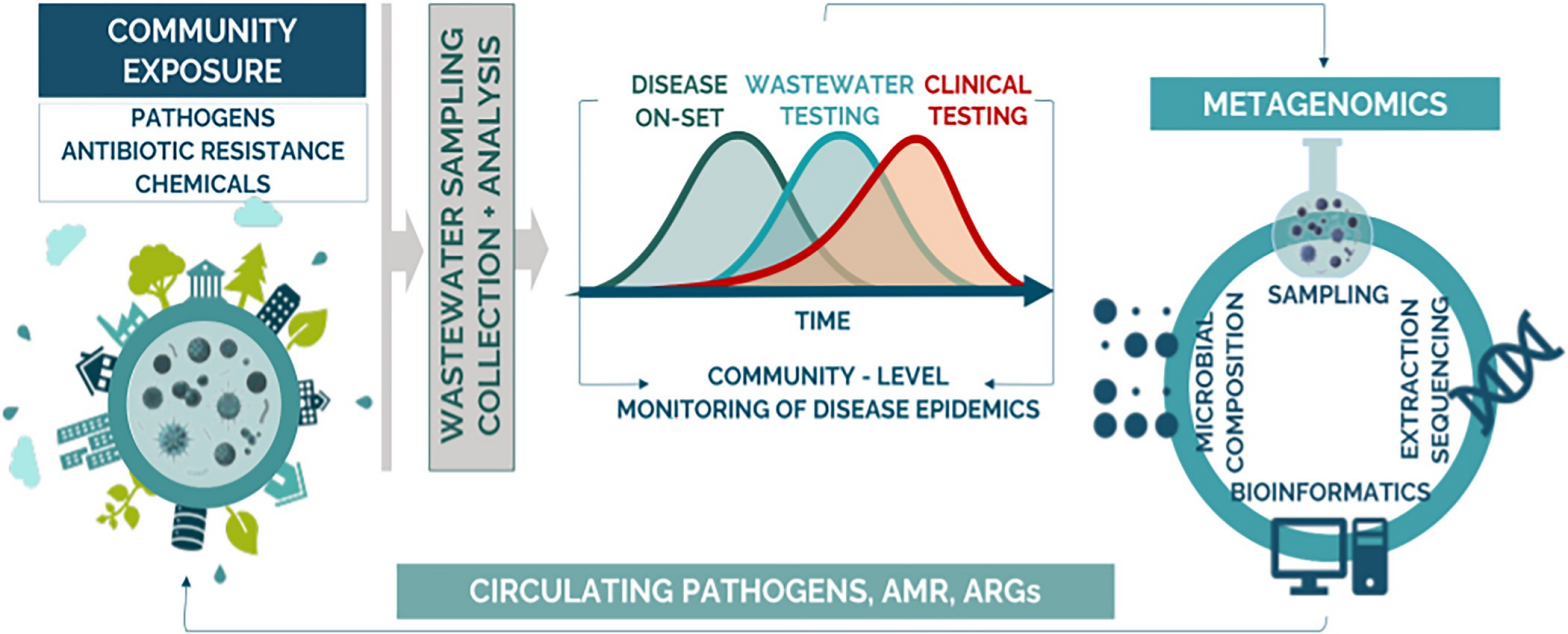
Metagenomics wastewater surveillance workflow.

Surveillance of hospital wastewater provides a proactive method for the early detection of outbreaks with epidemic and pandemic potential. By analyzing wastewater from healthcare facilities, public health officials can identify the presence of pathogens before patients exhibit symptoms, facilitating rapid intervention and containment measures (**see Fig 2).** This approach can detect a broad spectrum of pathogens, including viruses, bacteria, and fungi, offering a comprehensive view of potential health threats circulating within a community. Moreover, WWS acts as a non-invasive, cost-effective tool that complements traditional surveillance methods, thereby enhancing the capacity of health systems to respond effectively to infectious disease outbreaks.

Hospital wastewater is a critical reservoir for antimicrobial resistance due to the misuse of antibiotics, antibiotic residues, and fecal contamination. It is a source of multidrug-resistant bacteria, with secondary and tertiary healthcare centers contributing the most contaminated wastewater [21].

Studies in Burkina Faso, Benin, and Finland revealed regional differences in antibiotic resistance genes (ARGs), with Benin showing the highest abundance and Finland the lowest. Benin’s hospital wastewater was also identified as a significant reservoir for ESBL-producing *E*. *coli* [22]. Similar findings in Ghana highlighted the amplifying effects of hospitals on multidrug-resistant bacteria levels, with these contaminants entering urban sewage systems and posing health risks to both humans and animals [23]. A 2023 meta-analysis confirmed the health threats posed by ARGs and multidrug-resistance genes in hospital wastewater resistomes, emphasizing the need for robust wastewater management and surveillance [24].

### 2.10. Vaccine preventable diseases surveillance

**Fig 3:**
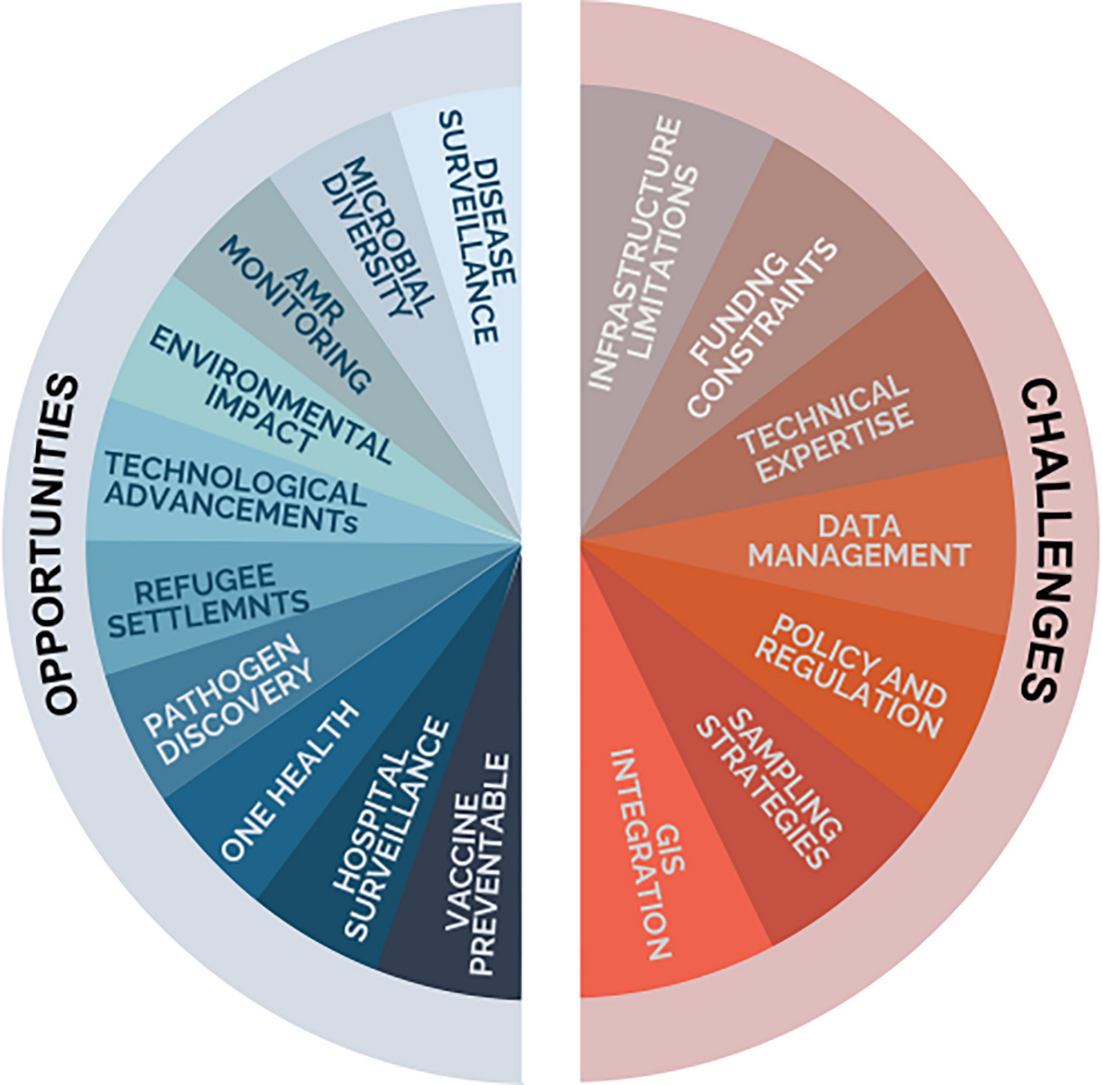
Opportunities and challenges of metagenomics surveillance in Africa.

Africa has a high rate of vaccine-preventable diseases (VPDs) such as cholera, hepatitis, measles, meningococcal meningitis, pneumonia, polio, rotavirus, rubella, tetanus, typhoid fever, yellow fever [25] and these require effective surveillance to ensure their eradication. Wastewater surveillance has proven invaluable for monitoring vaccine-preventable diseases and has been utilized by many countries to document elimination of enteroviruses [26] and detection of other emerging viruses [27]. It has demonstrated capacity as a non-invasive method to assess vaccination coverage and detect outbreaks, particularly in underserved areas [28]. The COVID-19 pandemic further demonstrated the usefulness of wastewater surveillance for outbreak identification since it allowed for prompt public health interventions even in situations where clinical testing was scarce by providing early indications of SARS-CoV-2 spread [29, 30]. Similarly, monitoring monkeypox in sewage offers a critical tool for tracking the virus in communities, especially where testing resources are scarce [31]. Therefore, integration of wastewater surveillance on top of other routine surveillance mechanisms can significantly improve disease detection and ensure prompt response to public health threats (**see Fig 3**).

## 3. Challenges

### 3.1. Infrastructure limitations

The impact of genomic surveillance on public health has become widely accepted [32]. Despite some progress, Africa continues to face challenges in developing its pathogen genomics infrastructure due to a variety of factors. These include a shortage of expertise, sustainable funding, and supply chain issues, as well as the high costs associated with operating and upgrading sequencing platforms. Additionally, there is a lack of technology resources, including computers and machines necessary for analysis, and difficulties in maintaining such equipment. These limitations compromise the quality of data collected, exacerbate training gaps, and contribute to the emigration of qualified personnel to other countries. Furthermore, infrastructure constraints significantly hinder the implementation of wastewater metagenomics in Africa.

Many public health laboratories lack essential high-throughput sequencing (HTS) facilities and advanced computational resources for analyzing complex environmental metagenomic data [12]. Despite progress during the COVID-19 pandemic, access to necessary HTS platforms and bioinformatics facilities remains limited and concentrated in a few countries [33]. Under these conditions, metagenomics wastewater surveillance can be significantly restrictive in terms of detection capabilities; sensitivity is reduced, and the scope is narrower, which often leads to extended turnaround times and delays in responding to disease outbreaks. Additionally, without reliable genomic data, it becomes challenging to conduct accurate epidemiological assessments or effectively plan for public health needs, especially given the limited collaboration among research communities. Overcoming these limitations could unlock the substantial potential of metagenomics surveillance. Moving forward, efforts should focus on leveraging established structures to enhance infrastructure and capacity-building programs. Additionally, developing policy frameworks that improve pathogen genomics and detection methods in wastewater surveillance will be crucial.

### 3.2. Funding constraints

One of the major obstacles to implementing wastewater metagenomics in Africa is the lack of adequate funding. The high costs associated with advanced sequencing technologies, specialized equipment, and skilled personnel present significant barriers for many research institutions and public health agencies across the continent. Additionally, sustained funding is crucial to support long-term surveillance programs that are essential for monitoring trends in pathogen emergence and antimicrobial resistance. The scarcity of financial resources restricts the capacity for conducting large-scale studies and impedes the development of robust metagenomic infrastructures. This challenge is compounded by the competitive nature of limited research grants, which may prioritize traditional public health initiatives over metagenomic projects. Addressing these funding challenges will require not only increased investment from national governments and international donors but also the development of innovative financing models to ensure the sustainability of wastewater metagenomics programs in Africa.

### 3.3. Technical expertise

The need for skilled personnel in genomics and bioinformatics is essential for the appropriate results interpretation. This helps in accurate identification, characterization and understanding of pathogens which are important for effective disease surveillance, outbreak response and critically serves for data-driven decision making and public health interventions. Africa hosts several bioinformatics hubs, such as the African Center of Excellence for Bioinformatics in Mali and Uganda and the H3Africa Bioinformatics Network, which support capacity building and research in genomics. Despite their contributions, these centers are relatively few to serve the whole continent. Challenges and limitations experienced with respect to this in Africa are tightly linked to the lack of trained personnel, the limited computational infrastructure, the poor access to necessary software and databases leading to the inability to process huge genomic datasets among others. Besides, data quality and standardization issues might lead to unreliable results. To bridge existing gaps, workshops and fellowships supported by organizations like H3Africa, the African Society for Bioinformatics and Computational Biology (ASBCB), Africa CDC, and international partners have been implemented. These efforts aim to enhance local expertise in sequencing and bioinformatics, but more targeted programs focusing on wastewater metagenomics are needed. These developments can reinforce global pathogen surveillance and could integrate more specific topics supported by genomics such as AMR tracking [34]. Nevertheless, it is important to standardize pipelines and databases as well as phenotypic predictions based on this information. Additionally, adoption of policies that enhance data sharing as well as collaboration can increase the ability for conducting genomics/metagenomic research. These studies show how important it is to develop bioinformatics capacity for better health [35], but also highlights the significance of global cooperation in advancing genomics research located at low-resource settings [34].

### 3.4. Data management

The vast amounts of data generated by metagenomics present a significant challenge in terms of data management. Effective data handling requires sophisticated bioinformatics tools and high-performance computing resources, which may not be readily available in many African research settings and public health institutions. Moreover, the lack of standardized protocols for data processing, storage, and sharing complicates the integration of metagenomic data into broader public health frameworks. Ensuring data quality, consistency, and security is crucial for the successful application of metagenomics in monitoring public health threats. However, many institutions in Africa face difficulties in managing these large datasets, which can limit the potential for collaborative research and data sharing across regions. To overcome this challenge, there is a need for investment in bioinformatics training, infrastructure development, and the establishment of data-sharing networks that can facilitate the effective use of metagenomic data for public health decision-making.

### 3.5. Policy and regulation

The rapid advancement of metagenomics has outpaced the development of ethical frameworks for the use and sharing of metagenomic data. Currently, there is a lack of comprehensive policies that adequately address the privacy, consent, and security concerns associated with such data. This gap poses significant risks, including potential misuse of sensitive information and inequitable access to data. Without robust regulatory frameworks, international collaboration is hindered, and the ethical integrity of research is compromised [36].

### 3.6. Sampling strategies

Developing effective sampling strategies is another critical challenge in the application of wastewater metagenomics in Africa. The heterogeneity of wastewater sources, varying environmental conditions, and logistical challenges in sample collection make it difficult to obtain representative and reliable samples. In many African settings, the infrastructure for systematic wastewater sampling is underdeveloped, leading to inconsistencies in sample quality and coverage. Additionally, the temporal and spatial variability of microbial communities in wastewater necessitates carefully designed sampling protocols to capture meaningful data. The lack of standardized sampling methods also hampers the comparability of results across different studies and regions. Addressing these challenges requires the development of context-specific sampling strategies that account for the unique environmental and infrastructural conditions in Africa. Collaboration between local researchers, public health authorities, and international experts can help establish best practices and optimize sampling efforts to enhance the reliability and impact of wastewater metagenomics in the region.

### 3.7. GIS integration

Geographic Information Systems (GIS) are essential for optimizing wastewater surveillance efforts on the African continent. Integrating spatial data with wastewater analysis allows for a strategic selection of sampling sites, tracking pathogen movement and informing targeted interventions. A study in Malawi used GIS to identify wastewater confluence points which formed the basis for site selection. Later, they classified high-density areas using GIS-based mapping and prioritized them for environmental surveillance. This targeted sampling method allowed for more efficient identification of sites, leading to the holistic representation of a community’s health [37]. Despite the immense potential of GIS, integrating it with existing systems presents significant challenges. Firstly, there is limited access to GIS infrastructure, with high costs associated with acquiring different licenses and developing spatial decision support systems. Secondly, many organizations lack access to personnel with the necessary skills for GIS. This leads to reliance on external assistance which limits local capacity building, leaving them unskilled and dependent on external help in the future as well. Thirdly, there is limited access to good quality, spatially referenced data. This is complicated by privacy concerns and data ownership restrictions. Moreover, there is inconsistency in data collection methods and reporting metrics across regions, which hinders comparative analysis [38].
