# Correction: Wastewater metagenomics in Africa: Opportunities and challenges

**DOI:** 10.1371/journal.pgph.0005097

**Published:** 2025-08-20

**Authors:** Stephen Kanyerezi, Fatma Zahra Guerfali, Abbas Abel Anzaku, Oluwasegun Adesina Babaleye, Tracey Calvert-Joshua, Julien Alban Nguinkal, Oluwaseun Paul Amoo, Chiraz Atri, Waqasuddin Khan, Iqra Saleh, M. Imran Nisar, Arthur Shem Kasambula, Koketso Morapedi, Gerald Mboowa

The following information is missing from the Funding statement: The Bill & Melinda Gates Foundation supported the establishment and work of the PHA4GE consortium.

The following statement is not indicated in the author byline: On behalf of the Public Health Alliance for Genomic Epidemiology (PHA4GE) Bioinformatics Pipelines and Visualization, Training and Workforce Development, and Data Repositories working groups.
